# Mediating role of birth at a baby-friendly hospital in the association between parental socioeconomic status and infant exclusive breastfeeding at six months old

**DOI:** 10.1186/s12889-023-17586-4

**Published:** 2024-01-03

**Authors:** Hong Mei, Yuanyuan Zhang, Ruixia Chang, Ke Xu, Jianduan Zhang, Fang Wang

**Affiliations:** 1 Institute of Maternal and Child Health, Tongji Medical College, Wuhan Children’s Hospital (Wuhan Maternal and Child Healthcare Hospital, Huazhong University of Science and Technology, 100 Hongkong Road, Wuhan, Hubei China; 2 Department of Maternal and Child Health, School of Public Health, Tongji Medical College, Huazhong University of Science and Technology, No.13, Hangkong Road, Wuhan, Hubei China; 3 Department of Obstetrics, Tongji Medical College, Wuhan Children’s Hospital (Wuhan Maternal and Child Healthcare Hospital, Huazhong University of Science and Technology, 100 Hongkong Road, Wuhan, Hubei China

**Keywords:** Baby-friendly hospital, Breast feeding, Socioeconomic status, Association

## Abstract

**Background:**

Current evidence suggests that the exclusive breastfeeding (EBF) rate at six months postpartum in China falls considerably below the targets recommended by the World Health Organization (WHO). Socioeconomic disparities in EBF have been observed in developing countries, with significant heterogeneity across studies. Despite the implementation of the Baby-Friendly Hospital Initiative (BFHI) in China since the 1990s to promote breastfeeding, there has been a lack of assessment concerning infants from different socioeconomic backgrounds. This study sought to investigate the association between socioeconomic status (SES) and EBF and explore the potential impact of giving birth at a Baby-Friendly Hospital (BFH) on this association.

**Methods:**

We analyzed data from 98,469 mother-child dyads selected from the Maternal and Child Health Management Information System. We used log-binomial models to examine the relationships between SES and EBF, SES and giving birth at a BFH, as well as BFH births and EBF. Additionally, we explored a counterfactual mediation approach to assess the mediating role of BFH births in the SES-EBF association.

**Findings:**

We identified a significant association between SES and EBF (RR_Medium vs. Low_ = 1.47, 95% CI 1.39–1.55; RR_High vs. Low_ = 1.40, 95% CI 1.32–1.49). Mothers with higher SES were more likely to give birth at BFHs (RR_Medium vs. Low_ = 1.85, 95% CI 1.81–1.88; RR_High vs. Low_=2.29, 95% CI 2.25–2.33). The significance of the SES-EBF association was attenuated when the type of hospital for childbirth was considered, revealing the significant mediating effect of BFH births in the SES-EBF association.

**Conclusion:**

Socioeconomic disparities are linked to infant EBF rates, with giving birth at a BFH mediating this association, especially for cases with low SES in rural areas.

**Supplementary Information:**

The online version contains supplementary material available at 10.1186/s12889-023-17586-4.

## Introduction

Breastfeeding offers significant benefits to both maternal and pediatric health, including the reduction of neonatal morbidity and mortality in the short term and a decreased risk of chronic illnesses such as childhood obesity, type 2 diabetes, and maternal breast and ovarian carcinoma in the long term [1]. The World Health Organization (WHO) recommends exclusive breastfeeding (EBF) for the first six months of an infant’s life [2]. However, the global prevalence of EBF remains suboptimal, with only 41% of infants being breastfed exclusively for the first six months in 2019 [3]. In China, the situation is even more concerning. According to a national survey conducted in 2013, the prevalence of EBF for infants under six months was only 20.7% [4], falling short of the WHO’s recommended target of achieving at least 50% EBF by 2025 [5].

Over the years, socioeconomic disparities in EBF have been extensively documented in various studies within developed countries. For example, a Norwegian population found a positive correlation between maternal education level and infant EBF practice in the fifth month after birth [6], a pattern also observed in a Belgian study [7]. In a large UK sample, mothers in higher managerial and professional occupations were more likely to exclusively breastfeed their children at four months of age compared to those engaged in routine jobs [8]. However, in some developing countries such as Ethiopia and Lebanon, a negative association between maternal education and EBF has been reported, with government-employed mothers less likely to breastfeed their infants [9]. China’s vast geographical expanse encompasses distinct regions with varying socioeconomic conditions [10]. Giving these difference in socioeconomic conditions and lifestyles, the relationship between socioeconomic status (SES) and EBF appears to be more complex, with different studies reporting positive, negative, and null associations [4, 11, 12]. In this respect, some studies indicated that mothers engaged in casual or agriculture-related jobs were more likely to practice EBF compared to professionals, white-collar workers, or those in industry and business-related fields [13]. Chen et al. even identified an inverted U-shaped relationship between maternal occupational status and infant breastfeeding duration [14]. Moreover, while maternal SES indices have been studied, limited evidence is available on the impact of paternal educational and occupational class on EBF. Given the multi-dimensionality and complexity of familial SES [15], studies that evaluate SES using both maternal and paternal socioeconomic information are necessary.

The Baby-Friendly Hospital Initiative (BFHI), an international approach to promoting breastfeeding has been implemented in China since the 1990s [16]. Hospitals following the ten steps of the BFHI provide mothers with greater access to professional feeding-related education, resulting in longer durations of EBF [17]. A previous meta-analysis demonstrated that BFHI interventions increased the likelihood of EBF for the first six months by 5.21-fold [18]. However, this meta-analysis did not include any studies from mainland China. As reported by Chen, et al., despite significant advancements over the past decade following the major healthcare reform initiated in 2009, China still faces disparities in various healthcare aspects [19]. For instance, health literacy, a well-documented concern, was found to be at an adequate level in only 22.3% of 3482 individuals sampled from 25 provinces in China, in stark contrast to a 72.3% adequacy rate in Japan [20]. The role of baby-friendly hospital (BFH), which implements BFHI interventions within the healthcare reform framework, in promoting EBF remains underexplored in China [21]. It has been observed that, compared to urban residents, those living in rural areas or a distance from major cities often encounter difficulties accessing BFHs [22]. Additionally, individuals with higher SES may prefer giving birth at prestigious hospitals, which are more likely to be BFHs [23, 24]. Accordingly, it is highly conceivable that a complex interplay exists among SES, giving birth at BFHs, and EBF. For example, giving birth at a BFH may potentially mediate the association between SES and EBF, necessitating further investigation.

Wuhan, a prominent megacity located in central China, boasts a registered population of approximately 11.08 million residents across 13 administrative districts as of 2017. These districts are further categorized into 7 central districts and 6 outlying districts. Of the 28 BFHs in Wuhan in 2016, only 3 were located in the outlying districts (Table S1). Given the socioeconomic disparities and variances in BFH accessibility within the healthcare landscape, in the current study, utilizing data from the Wuhan Maternal and Child Health Management Information System (WMCHMIS) [25], we aim to address the following key questions: First, is there a correlation between SES and infant EBF at six months of age; Second, is there a relationship exist between SES and access to BFHs in the Chinese population under study? Thirdly, does giving birth at a BFH mediate the SES-EBF association?

## Methods and material

### Participants and study design

Following a prospective cohort study design, participants for this study were selected from the WMCHMIS, which was established in 2003 for public health surveillance, and has been used for research purposes since 2010 [25]. The WMCHMIS continually updates primary care data, encompassing maternal health check-ups, delivery data, postnatal care information, and child health data from birth to six years old, originating from maternal and child health care institutions throughout Wuhan. For this study, we selected a total of 98,469 mother-child dyads from the WMCHMIS covering the years 2011 to 2017. Inclusion criteria were as follows: (1) maternal age at childbirth ≥ 18 years, (2) live birth of singleton pregnancy without visible congenital disabilities, and (3) the available of mother-infant dyads information, including familial socioeconomic status, infant feeding patterns at six months old, maternal pre-pregnancy BMI, gestational weight gain, birth mode, and neonatal birth weight. Notably, China initiated its universal two-child policy in January 2016. To mitigate the influence of having multiple children within a family and prior infant feeding experiences, and to maintain consistency in the study data spanning from 2011 to 2017, we added an inclusion criteria specifying primipara status (i.e., mothers giving birth for the first time without experiencing miscarriage after 28 gestational weeks).

### Definition of EBF and BFH

Data on infant feeding patterns at six months old were collected during the routine 6-month health check-up (usually conducted at 6 to 7 months of age). Trained nurses explained the various feeding types (exclusive breastfeeding, any breastfeeding, mixed feeding, or formula feeding) to the child’s caretaker and inquired about the specific feeding pattern the infant had been under during the first six months of life. In this study, we categorized the feeding pattern into two groups: EBF and non-EBF. EBF was defined in accordance with the WHO criteria, whereby the infant received only human breast milk except for oral rehydration solution, vitamin drops, syrups for essential nutrients, or necessary medicines, while refraining from additional foods or drinks, including water [26].

The names of hospitals were extracted from the WMCHMIS and were classified as either BFH or non-BFH based on the BFH list provided by the National Health and Family Planning Commission (Table S1).

### Socioeconomic status

Given the high proportion of missing data for family income (approximately 77%), we focused on four SES indices encompassing the educational levels and occupations of both mothers and fathers. Occupational categories included agriculture and forestry, laborer, service staff, office clerk, professional & technical personnel, managerial workers, soldiers, and unemployed. Educational levels were grouped into four categories based on the number of schooling years completed: less than high school, high school, college or university, and postgraduate or above. Educational level and occupational type were rated according to established methodologies (Table S2) [27, 28]. Parental occupations were then classified into low-prestige (score ≤ 49) and high-prestige (score > 49) groups (Table S2). The overall SES score was calculated as follows: (0.7 * (maternal education + paternal education) + 0.4 * (maternal occupation + paternal occupation))/2 [27, 29]. Overall SES was subsequently categorized as Low, Medium, and High groups according to tertiles (with cutoff points set at 33.3% and 66.6%).

### Confounding factors

Maternal pre-pregnancy BMI was calculated by dividing weight by height^2^ (kilograms per meter squared) and categorized as underweight (BMI < 18.5 kg/m^2^), normal weight (18.5 kg/m^2^ ≤ BMI < 24 kg/m^2^), and overweight/obese (BMI ≥ 24 kg/m^2^), following the guidelines of the Working Group on Obesity in China [30]. Gestational weight gain, representing the difference in maternal weight before pregnancy and at birth, was classified in accordance with the guidelines provided by the Chinese Nutrition Society [31]. Birth mode of delivery was categorized as transvaginal or cesarean section. Living areas were classified as urban or rural, depending on the permanent address of the families. Families residing in the central districts were classified as urban, while those residing in the outlying districts were categorized as rural.

### Statistical analyses

Categorical variables were described using frequency and proportion. The *chi-square* test was used to assess the difference between category variables. The log-binomial model was employed to investigate the associations between SES and EBF, SES and BFH access, and BFH and EBF, with risk ratios (RRs) and 95% confidence intervals (CIs) reported. Additionally, an interaction effect analysis was conducted to assess the interaction between SES and hospital type (BFH or non-BFH) on EBF.

To examine the mediating role of giving birth at a BFH in the SES-EBF association, a counterfactual mediation approach was applied. The total effect was deconstructed into natural direct and indirect effects in a simple mediation model. When considering the exposure-mediator interaction (SES-BFH), the direct effect was denoted as both pure and total direct effects, and the indirect effect was similarly represented as pure and total indirect effects. In our study, the pure direct effect indicated the increased likelihood of EBF at six months old when SES changed from the reference (Low) to the comparison group (Medium/High) for an infant while controlling for covariates and the hospital type at the level naturally observed in the SES reference group. The definition of total direct effect closely mirrored the concept of pure direct effect, but with hospital type controlled at the level naturally observed in the SES comparison group. The pure indirect effect indicated the increased likelihood of EBF at six months old when hospital type transitioned from the level as naturally observed in the reference group (Low) to the level as naturally observed in the comparison group (Medium/High) while controlling for covariates and SES at the reference group level. The total indirect effect was defined similarly to the pure indirect effect, except that SES was fixed at the comparison group level. The relative risk (RR) for the total effect was computed by multiplying the RRs for the pure direct effect, pure indirect effect, and the mediated interaction RR. This calculation adhered to the expressions for primary and interaction terms in linear regression, and specific details regarding these effects were referenced from the *medflex* package [32].

Sensitivity analyses were conducted for all analyses based on living areas. All analyses were conducted using R software, and the mediation analysis was performed using the *medflex* package [32]. A *P-value* < 0.05 was statistically significant.

## Results

### Characteristic description

The flowchart of our study is depicted in Figure S1. We included 98,469 mother-child dyads in the final analysis, with 11,612 (11.8%) infants exclusively breastfed at six months postpartum. The characteristics of these dyads are summarized in Table 1. Out of the total 59,503, 60.4% of mothers gave birth in BFHs, and the EBF rate for their infants at six months was 15.2%. In contrast, among 38,966 (39.3%) infants born outside BFHs, the EBF rate was notably lower (6.7%). Infants with parents possessing a higher educational level, holding prestigious occupations, or having higher comprehensive SES status were more likely to be exclusively breastfed (Table 1). Additionally, compared to those living in rural areas, parents in urban areas exhibited higher educational levels, occupational prestige, overall SES, and great access to BFHs (Table S3).

**Table 1:** Basic characteristics of 98,469 mother-child dyads

Variables	Class	NEBF [n (%), N = 86,857]	EBF [n (%), N = 11,612)	Total (n, N = 98,469)
Maternal education^*^	≤ 9 y	35,052 (90.3)	3,757 (9.7)	38,809
	10–12 y	15,025 (88.7)	1,917 (11.3)	16,942
	13–15 y	14,957 (86.8)	2 277 (13.2)	17,234
	≥ 16 y	21,823 (85.6)	3,661 (14.4)	25,484
Paternal education^*^	≤ 9 y	19,638 (93.4)	1,390,(6.6)	21,028
	10–12 y	22,986 (88.7)	2,914,(11.3)	25,900
	13–15 y	17,087 (85.5)	2,887,(14.5)	19,974
	≥ 16 y	27,146 (86.0)	4,421,(14.0)	31,567
Maternal occupation^*^	Low prestige	56,975 (90.6)	5,908,(9.4)	62,883
	High prestige	29,882 (84.0)	5,704,(16.0)	35,586
Paternal occupation^*^	Low prestige	53,816 (91.0)	5,297,(9.0)	59,113
	High prestige	33,041 (84.0)	6,315,(16.0)	39,356
Parental occupation^*^	Parental low prestige	48,196 (91.5)	4,489 (8.5)	52,685
	Maternal low and paternal high prestige	8,779 (86.1)	1,419 (13.9)	10,198
	Maternal high and paternal low prestige	5,620 (87.4)	808 (12.6)	6,428
	Parental high prestige	24,262 (83.2)	4,896 (16.8)	29,158
Overall SES^*^	Low	30,015 (92.5)	2,446 (7.5)	32,461
	Medium	29,332 (86.9)	4,440 (13.1)	33,772
	High	27,510 (85.3)	4,726 (14.7)	32,236
Maternal childbearing age^*^	< 25 y	19,394 (91.0)	1,916 (9.0)	21,310
	25-<30 y	47,642 (88.1)	6,465 (11.9)	54,107
	30-<35 y	16,664 (86.2)	2,663 (13.8)	19,327
	≥ 35 y	3,157 (84.8)	568 (15.2)	3,725
Maternal pre-pregnancy BMI^*^	Underweight	19,432 (89.5)	2,270 (10.5)	21,702
	Normal	61,190 (87.9)	8,418 (12.1)	69,608
	Overweight/ Obesity	6,235 (87.1)	924 (12.9)	7,159
Gestational weight gain^*^	Excessive	56,200 (88.6)	7,264 (11.4)	63,464
	Inadequate	4,545 (87.6)	642 (12.4)	5,187
	Appropriate	26,112 (87.6)	3,706 (12.4)	29,818
Infant birth weight	< 2500 g	780 (88.1)	105 (11.9)	885
	2500–4000 g	81,504 (88.2)	10,874 (11.8)	92,378
	≥ 4000 g	4,573 (87.8)	633 (12.2)	5,206
Birth mode^*^	Transvaginal	33,583 (86.2)	5,382 (13.8)	38,965
	Cesarean	53,274 (89.5)	6,230 (10.5)	59,504
Birth at a baby-friendly hospital^*^	No	36,372 (93.3)	2,594 (6.7)	38,966
	Yes	50,485 (84.8)	9,018 (15.2)	59,503
Sex^*^	Girl	40,814 (87.9)	5,639 (12.1)	46,453
	Boy	46,043 (88.5)	5,973 (11.5)	52,016
Living area^*^	Urban	38,213 (82.6)	8,033 (17.4)	46,246
	Rural	44,102 (94)	2,835 (6)	46,937

^*^: *p* values significant (< 0.05)

BMI: body mass index; EBF: exclusive breastfeeding; NEBF: not exclusively breastfeeding; SES: socioeconomic status

### The association between SES and EBF

Table 2 presents the relationship between SES and EBF. Parental education was positively associated with EBF in both Model 1 (unadjusted) and Model 2 (adjusted for covariates). However, when we further adjusted for birth at a BFH (Model 3), the association between maternal education (except for ≥ 16y vs. ≤ 9y) and EBF was no longer significant. All three models consistently indicated that infants born to parents with high-prestige occupations were more likely to be breastfed compared to those with low-prestige occupations. Compared with results from urban populations, results from rural areas were more in line with those in Table 2 (Table S4).

**Table 2:** Association between SES and EBF at six months old

Variable	Class	Model 1^a^ OR (95% CI)		Model 2^b^ OR (95% CI)		Model 3^c^ OR (95% CI)
Maternal education	≤ 9 y	REF		REF		REF
	10–12 y	1.17 (1.11, 1.23) ^*^		1.16 (1.10, 1.22) ^*^		1.03 (0.98, 1.08)
	13–15 y	1.36 (1.30, 1.43) ^*^		1.28 (1.22, 1.35) ^*^		1.04 (0.99, 1.09)
	≥ 16 y	1.48 (1.42, 1.55) ^*^		1.33 (1.27, 1.39) ^*^		1.05 (1.00, 1.10) ^*^
Paternal education^*^	≤ 9 y	REF		REF		REF
	10–12 y	1.70 (1.60, 1.81)		1.65 (1.56, 1.76)		1.43 (1.34, 1.52)
	13–15 y	2.19 (2.06, 2.32)		2.05 (1.92, 2.18)		1.59 (1.49, 1.69)
	≥ 16 y	2.12 (2.00, 2.24)		1.93 (1.82, 2.05)		1.45 (1.47, 1.55)
Maternal occupation^*^	Low prestige	REF		REF		REF
	High prestige	1.71 (1.65, 1.77)		1.59 (1.53, 1.64)		1.31 (1.26, 1.36)
Paternal occupation^*^	Low prestige	REF		REF		REF
	High prestige	1.79 (1.73, 1.85)		1.68 (1.62, 1.74)		1.40 (1.35, 1.45)
Parental occupation^*^	Parental low prestige	REF		REF		REF
	Maternal low and paternal high prestige	1.63 (1.54, 1.73)		1.58 (1.49, 1.67)		1.42 (1.33, 1.51)
	Maternal high and paternal low prestige	1.48 (1.38, 1.58)		1.42 (1.32, 1.52)		1.228 (1.12, 1.32)
	Parental high prestige	1.97 (1.90, 2.05)		1.84 (1.77, 1.92)		1.58 (1.51, 1.66)
Overall SES^*^	Low	REF		REF		REF
	Medium	1.74 (1.66, 1.83)		1.68 (1.60, 1.76)		1.47 (1.39, 1.55)
	High	1.95 (1.86, 2.04)		1.78 (1.69, 1.87)		1.40 (1.32, 1.49)

CI: confidence interval; OR: odds ratio; SES: socioeconomic status. ^*^: *p* values for all levels significant

^a^Model 1 used a log-binomial model without covariates

^b^Model 2 used a log-binomial model with covariates including maternal childbearing age, maternal pre-pregnancy BMI, gestational weight gain, infant birth weight, birth mode, and sex

^c^Model 3 used a log-binomial model with covariates including variables adjusted in model 2 and birth at a baby-friendly hospital

### The association between SES and BFH

Table 3 outlines the relationship between SES and birth at a BFH. Parental educational level and occupational prestige were positively associated with giving birth at a BFH. When considering comprehensive SES, the percentage of mothers exclusively breastfeeding in the low SES group was substantially lower than that in the high SES group, with 32.4% exclusively breastfeeding in the low SES group. Positive associations between SES and BFH access were evident in both the raw and adjusted log-binomial models (RR _medium_ = 1.85, 95% CI 1.81–1.88; RR _high_ = 2.29, 95% CI 2.25–2.33). Compared with rural populations, the effect of SES on BFH was more minor for urban populations (urban area RR _high_ = 1.20, 95% CI 1.18–1.22; rural area RR _high_ = 2.25, 95% CI 2.18–2.32) (Table S5).

**Table 3:** Association between SES and birth at a baby-friendly hospital

Variable	Class	Non-BFH [n (%)]	BFH [n (%)]	Total (n)	Model 1^a^ OR (95% CI)		Model 2^b^ OR (95% CI)
Maternal education^*^	≤ 9 y	23,310 (60.1)	15,499 (39.9)	38,809	REF		REF
	10–12 y	7,363 (43.5)	9,579 (56.5)	16,942	1.42 (1.39, 1.44)		1.31 (1.29, 1.33)
	13–15 y	4,278 (24.8)	12,956 (75.2)	17,234	1.88 (1.85, 1.91)		1.57 (1.55, 1.59)
	≥ 16 y	4,015 (15.8)	21,469 (84.2)	25,484	2.11 (2.08, 2.14)		1.66 (1.64, 1.68)
Paternal education^*^	≤ 9 y	15,005 (71.4)	6,023 (28.6)	21,028	REF		REF
	10–12 y	12,552 (48.5)	13,348 (51.5)	25,900	1.80 (1.76, 1.84)		1.73 (1.69, 1.77)
	13–15 y	5,391 (27.0)	14,583 (73.0)	19,974	2.55 (2.49, 2.61)		2.28 (2.23, 2.34)
	≥ 16 y	6,018 (19.1)	25,549 (80.9)	31,567	2.83 (2.76, 2.89)		2.43 (2.38, 2.49)
Maternal occupation^*^	Low prestige	33,706 (53.6)	29,177 (46.4)	62,883	REF		REF
	High prestige	5,260 (14.8)	30,326 (85.2)	35,586	1.84 (1.82, 1.85)		1.61 (1.60, 1.63)
Paternal occupation^*^	Low prestige	32,258 (54.6)	26,855 (45.4)	59,113	REF		REF
	High prestige	6,708 (17.0)	32,648 (83.0)	39,356	1.83 (1.81, 1.84)		1.61 (1.59, 1.63)
Parental occupation	Parental low prestige	30,789 (58.4)	21,896 (41.6)	52,685	REF		REF
	Maternal low and paternal high prestige	2,917 (28.6)	7,281 (71.4)	10,198	1.72 (1.69, 1.75)		1.61 (1.59, 1.64)
	Maternal high and paternal low prestige	1,469 (22.9)	4,959 (77.1)	6,428	1.86 (1.83, 1.89)		1.71 (1.68, 1.74)
	Parental high prestige	3,791 (13)	25,367 (87)	29,158	2.09 (2.07, 2.12)		1.84 (1.81, 1.86)
Overall SES^*^	Low	21,930 (67.6)	10,531 (32.4)	32,461	REF		REF
	Medium	12,400 (36.7)	21,372 (63.3)	33,772	1.95 (1.92, 1.99)		1.85 (1.81, 1.88)
	High	4,636 (14.4)	27,600 (85.6)	32,236	2.64 (2.60, 2.68)		2.29 (2.25, 2.33)

BMI: body mass index; CI: confidence interval; BFH: baby-friendly hospital; Non-BFH: not baby-friendly hospital; OR: odds ratio; SES: socioeconomic status

^*^: *p* values for all levels significant

^a^Model 1 used a log-binomial model without covariates

^b^Model 2 used a log-binomial model with covariates including maternal childbearing age, pre-pregnancy BMI, gestational weight gain and birth mode

### The association between BFH and EBF

According to the results of the adjusted log-binomial model (Table 4), infants born in BFHs had an increased likelihood of exclusively breastfeeding at six months of age (RR = 1.94, 95% CI 1.85–2.03). The BFH-EBF relationship was most pronounced in the Low SES group (RR = 3.04, 95% CI 2.80–3.29) compared to the Medium (RR = 1.61, 95% CI 1.51–1.72) and High (RR = 1.39, 95% CI 1.27–1.52) SES groups. The included covariates are familial comprehensive SES, maternal childbearing age, pre-pregnancy BMI, gestational weight gain, infant birth weight, birth mode, and sex. Giving birth at a BFH demonstrates a protective effect on EBF for rural populations (RR = 2.45, 95% CI 2.27–2.64), while it appears to be a risk factor for urban populations regarding EBF (RR = 0.91, 95% CI 0.86–0.96) (Table S6).

**Table 4:** Association between BFH and EBF

	Model 1^a^ OR (95% CI)	Model 2^b^ OR (95% CI)
Overall population^*^		
Non-BFH	REF	REF
BFH	2.13 (2.04, 2.23)	1.94 (1.85, 2.03)
Low SES group^*^		
Non-BFH	REF	
BFH	3.04 (2.80, 3.29)	
Medium SES group^*^		
Non-BFH	REF	
BFH	1.61 (1.51, 1.72)	
High SES group^*^		
Non-BFH	REF	
BFH	1.39 (1.27, 1.52)	

^*^: *p* values for all levels significant. BFH: baby-friendly hospital; Non-BFH: not baby-friendly hospital

^a^ Model 1 used a log-binomial model with covariates including maternal childbearing age, pre-pregnancy BMI, gestational weight gain, birth mode, infants’ sex and birth weight

^b^ Model 2 used a log-binomial model with covariates including overall socioeconomic status, maternal childbearing age, pre-pregnancy BMI, gestational weight gain, birth mode, infants’ sex, and birth weight

### The mediating role of BFH on the SES-EBF association

**Fig. 1:**
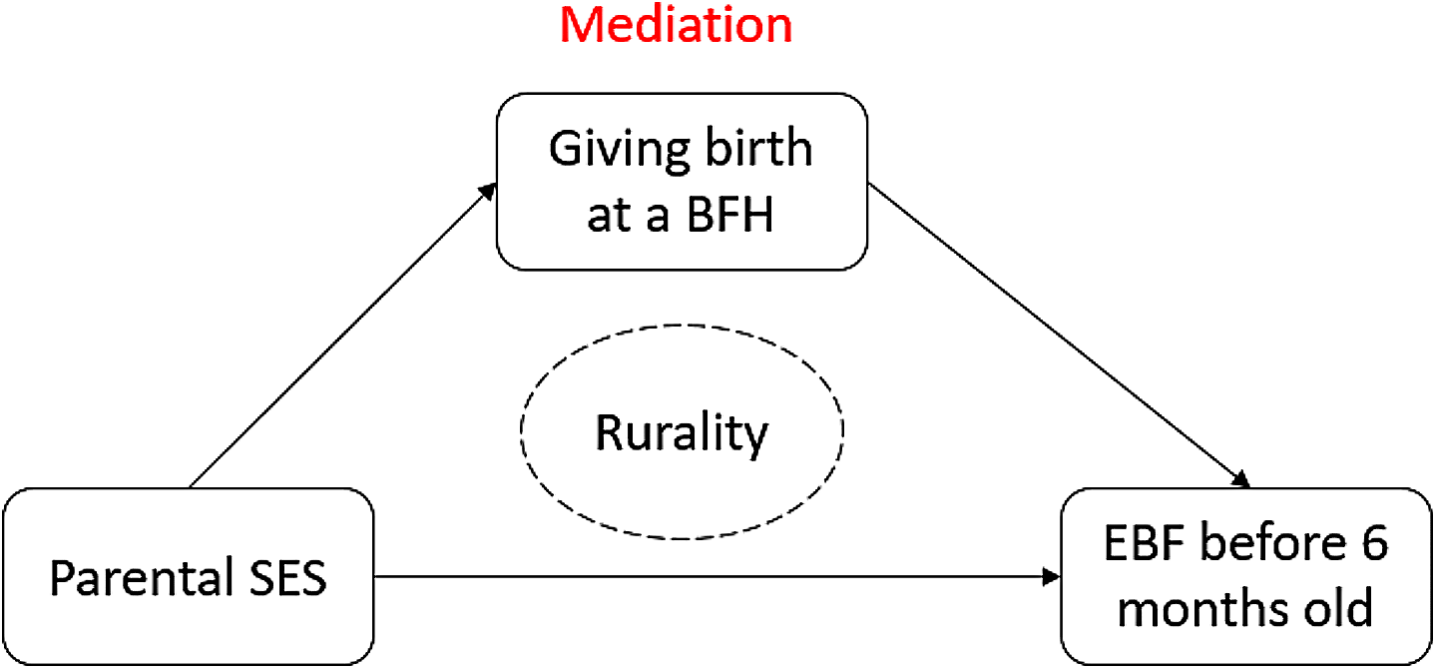
A quick diagram of association analysis among SES, BHF and EBF. SES, socioeconomic status; BFH, baby-friendly hospital; EBF, exclusive breastfeeding

During mediation analysis, considering the interaction effect between SES and hospital type (Fig. 1), we identified the mediating role of giving birth at a BFH in the SES-EBF association. For instance, as shown in Table 5, after controlling for covariates at baseline, the shift in SES in BFH from the Low to the High group resulted in a 1.58-fold [indirect effect, exp(0.46)] increase in the probability of EBF at six months postpartum. The mediation analysis also indicated that the impact of SES on EBF through giving birth at a BFH was more substantial for mothers with low SES compared to those with higher SES. For instance, when we adjusted for covariates at baseline, the shift in breastfeeding habits from the Low SES group to the High group resulted in only a 1.13-fold [indirect effect, exp(0.12)] increase in the likelihood for EBF at six months of age. Similar mediation associations were presented for both urban and rural populations (Table S7).

**Table 5:** The mediation effect of birth at a baby-friendly hospital in the association between familial socioeconomic status and infant exclusive breastfeeding at six months old

Groups^a^	Direct effect [log (RR)]			Indirect effect [log (RR)]		Total effect [log (RR)]
	Pure	Total		Pure	Total	
Medium vs. Low^*^	0.39 (0.03)	0.22 (0.03)		0.30 (0.01)	0.13 (0.01)	0.52 (0.02)
High vs. Low^*^	0.46 (0.03)	0.13 (0.03)		0.46 (0.01)	0.12 (0.02)	0.58 (0.03)

^a^Covariates in the mediation model included maternal childbearing age, pre-pregnancy BMI, gestational weight gain, infant birth weight, birth mode, and sex

^*^refers to *p* < 0.005 for all levels

The direct and indirect effects for all the comparing groups were significant among the direct (both pure and total effect), indirect (both pure and total effect) and total effects

## Discussion

The present study identified a positive association between SES with infant EBF at six months and SES with BFH and revealed that the mediating role of birth at a BFH played a crucial role in the SES-EBF association.

Over the past three decades, the EBF rate in China has experienced fluctuations due to changes in lifestyle and socioeconomic conditions. It decreased from 53.7% in 1998 to 20.8% in 2013 [33], and reached 29.2% by 2019 with the implementation of health promotion actions and education [34]. However, the EBF rate in our sample was only 12%, lower than the national rate in China, and fell far below the WHO’s recommended standards. The variations could be attributed to the difference in data sources used and the definitions of EBF [35]. In the present study, we defined EBF as the infant received only human breast milk except for oral rehydration solution, vitamin drops, syrups for essential nutrients, or necessary medicines, while refraining from additional foods or drinks, including water according to the WHO criteria, and the data was calculated based on oral-interview results recorded in the WMCHMIS by trained nurses. The strict definition and vigorous way of data collection may account for the comparatively low EBF rates. Moreover, Wuhan, a major city in central China with a population of over 11.08 million, may exhibit unique characteristics regarding EBF, influenced by various factors, which adds an intriguing dimension to our study.

Our findings are consistent with research conducted in developed countries [6, 7, 36], highlighting the positive relationship between maternal education and infant EBF prevalence at six months, especially for those residing in rural areas. It is widely believed that well-educated mothers have greater access to information and support for breastfeeding [7]. Similarly, positive associations were observed for parental occupational status, aligning with a study conducted by Chen et al. using data from the China Family Panel Studies [14]. We hypothesized that mothers with higher-prestige occupations might have more time and availability for exclusive breastfeeding, both from their families and employers [37]. Besides, the association between paternal SES and EBF has been underreported in China than in Western countries [14, 38, 39]. Since 2017, there has been widespread advocacy and discussion surrounding paternal-infant bonding in breastfeeding. Given that majority of our data was collected before 2017, it is highly conceivable that families in our study may not have had a comprehensive understanding of fathers’ role in breastfeeding [40–44]. Nonetheless, we identified a significant association between paternal SES and EBF, consistent with research utilizing data from the China Family Panel Studies [40–44]. Besides, we found that paternal educational level and occupation prestige had stronger associations with EBF compared with maternal educational level and occupation prestige. We inferred that fathers with higher education and higher-prestige occupation might have more access to the breastfeeding policy and publicity, and provide their wives with more emotional and financial support for breastfeeding, ultimately reducing the likelihood of cessation before six months [45]. Additionally, in a lot of scenarios, fathers’ decision is important in a household in traditional Chinese families [46], which might make the fathers’ influence bigger than mothers. Collectively, these findings emphasize the importance of considering paternal SES, in addition to maternal SES, in promoting exclusive breastfeeding.

Our study also identified socioeconomic inequalities associated with birth at BFHs. It has been established that in a provincial capital city in central China, more than 90% of BFHs are tertiary hospitals with adequate medical resources and solid medical strength. Generally, in the Chinese three-level medical system, i.e., primary, secondary, and tertiary hospitals, the higher the level of hospitals, the lower the reimbursement rate of free medical insurance, and the higher the hospitalization expenses would be [47, 48]. According to our study, people in the low SES group were less likely to choose birth at BFHs and prone to stop EBF before six months postpartum, which was in accordance with the findings reported by Kim and his colleagues [18].

During the first six months of an infant’s life, breastfeeding practices can be categorized into four stages: the preparatory stage before delivery, the initial stage in the hospital, the self-exploratory stage after discharge, and the transitional stage between postnatal months four and six [49]. During the initial stage, intentions for breastfeeding often decrease due to perceived insufficient breast milk, the discomfort of incorrect attachment techniques, and concerns about jaundice [49]. The training and supportive atmosphere for breastfeeding in hospitals can significantly promote the initiation of EBF and its continuation for extended period after discharge [17]. In our study, we also observed the association between BFH and EBF, especially in the low SES group and rural populations. Mothers with higher SES in urban areas typically have better access to qualified breastfeeding knowledge both before and during pregnancy [50], which might limit the impact of BFHI on EBF for this group. The variations in the BFH-EBF relationship across different SES levels underscore the importance of providing robust breastfeeding support, especially to those with low SES and in rural areas [14]. Besides, by employing a counterfactual mediation approach, we identified the mediating role of BFHI in the SES-EBF association. The mediation analysis also revealed a stronger association in the rural populations. In summary, to enhance EBF rates, our results underscore the significant impact of SES and the importance of ensuring access to BFHs, especially for those with low SES and in rural areas. Recognizing the pivotal role of birth at a BFH in promoting EBF, BFHI promotion programs targeting low socioeconomic and rural populations could yield substantial public health benefits [51]. Additionally, there is a need to formulate policies to ensure the effective implementation of EBF, such as making policies encouraging primary hospitals and health centers to achieve BFHI certification and expanding BFH to a larger segment of the population.

In addition to the main findings, we also found that covariates such as maternal pre-pregnancy BMI, gestational weight gain, maternal childbearing age and birth mode were associated with EBF (Table S8). Several studies reported the negative effect of obesity in mothers on EBF duration [52–54], while in our study, no association was found between normal weight and overweight/obese mothers. We found that compared with the pre-pregnancy underweight mothers, the normal weight and overweight/obese mothers were more inclined to EBF at six months of age, which was not previously reported. Moreover, excessive gestational weight gain, older maternal childbearing age and cesarean birth were risk factors for EBF at six months of age, which were in accordance with a previous Japanese study [51]. Nonetheless, further research is warranted to validate the findings of our study.

Several limitations of our study should be acknowledged. Firstly, obtaining precise and reliable income information in public places like hospitals was challenging. Consequently, the income variable was excluded from SES evaluation in our study due to a substantial proportion of missing values (77%). Secondly, our classification of parental occupations relied on a broad taxonomy, overlooking detailed information, particularly regarding maternal occupation, including factors like working hours, labor intensity, commute times, workplace environments, and employment benefits. Thirdly, the BFHs assessed in our study were evaluated in 2017, and the quality of hospital care may vary over the years covered by our data, spanning from 2011 to 2017. Furthermore, our retrospective study could not estimate improvements in individual breastfeeding skills over time. Fourthly, information on the knowledge about breastfeeding among family members, especially fathers, was not available in our study. This knowledge has been associated with SES and EBF behavior in several studies [55–58], and future research on the relationship between SES and EBF could consider including this variable. Finally, our analysis was limited to data from families of primiparous mothers, and further investigation is warranted to determine if the same associations hold for multiparous families.

## Conclusions

Our study, with an EBF rate of 12% at six months old, revealed that birth at a BFH mediated the association between SES and infant EBF practice at six month postpartum, especially for populations with low SES in rural areas. Collectively, our findings emphasized that policies are needed to ensure access to BFH in order to improve the EBF status, especially for those with low SES and living in rural areas. Besides, more attention should be paid to the fathers’ role in the implementation of EBF.

### Electronic supplementary material

Below is the link to the electronic supplementary material.

Supplementary Material 1

## Data Availability

The datasets used and/or analyzed during the current study are available from the corresponding author on reasonable request.
