# Supplementary material for: Mediating role of birth at a baby-friendly hospital in the association between parental socioeconomic status and infant exclusive breastfeeding at six months old
Source: BMC Public Health. 2024 Jan 3;24:78. doi: 10.1186/s12889-023-17586-4 (PMC10762853; doi:10.1186/s12889-023-17586-4)
Supplement: Supplementary file 1 — Supplementary Material 1 [file 12889_2023_17586_MOESM1_ESM.docx]

**Mediating role of birth at a baby-friendly hospital in the association between parental** **socioeconomic status and infant exclusive breastfeeding at six months old**

Table S1. The list of baby-friendly hospital

| Hospital | Location | Year  released | Year  evaluated | Year applied  in our data |
| --- | --- | --- | --- | --- |
| Tongji Hospital affiliated to Tongji Medical College of Huazhong University of Science & Technology | Central area | 2015 | 2011-2014 | 2011-2017 |
| Union Hospital affiliated to Tongji Medical College of Huazhong University of Science and Technology | Central area | 2015 | 2011-2014 | 2011-2017 |
| Zhongnan Hospital of Wuhan University | Central area | 2015 | 2011-2014 | 2011-2017 |
| Renmin Hospital of Wuhan University | Central area | 2015 | 2011-2014 | 2011-2017 |
| Tianyou Hospital affiliated to Wuhan university of Science and Technology | Central area | 2015 | 2011-2014 | 2011-2017 |
| Hubei Provincial Hospital of Traditional Chinese Medicine | Central area | 2015 | 2011-2014 | 2011-2017 |
| Maternal and Child Hospital of Hubei province | Central area | 2015 | 2011-2014 | 2011-2017 |
| Hubei Hospital of Integrated Traditional Chinese and Western Medicine | Central area | 2015 | 2011-2014 | 2011-2017 |
| General Hospital of Guangzhou Military Area Command of Wuhan | Central area | 2015 | 2011-2014 | 2011-2017 |
| Hubei Provincial Corps Hospital of Chinese People Armed Police Forces | Central area | 2015 | 2011-2014 | 2011-2017 |
| The 161st Hospital of the People's Liberation Army | Central area | 2015 | 2011-2014 | 2011-2017 |
| The 457st Hospital of the People's Liberation Army | Central area | 2015 | 2011-2014 | 2011-2017 |
| Wuhan Hospital of Integrated Traditional Chinese and Western Medicine | Central area | 2015 | 2011-2014 | 2011-2017 |
| The Central hospital of Wuhan | Central area | 2015 | 2011-2014 | 2011-2017 |
| Wuhan Children's Hospital | Central area | 2015 | 2011-2014 | 2011-2017 |
| Wuhan Changhang General Hospital | Central area | 2015 | 2011-2014 | 2011-2017 |
| Wuhan Eighth Hospital | Central area | 2015 | 2011-2014 | 2011-2017 |
| Hankou Hospital of Wuhan | Central area | 2015 | 2011-2014 | 2011-2017 |
| Puai Hospital affiliated to Tongji Medical College of Huazhong University of Science and Technology | Central area | 2015 | 2011-2014 | 2011-2017 |
| Wuhan Fifth hospital | Central area | 2015 | 2011-2014 | 2011-2017 |
| Hanyang Hospital of Wuhan | Central area | 2015 | 2011-2014 | 2011-2017 |
| Wuhan Third hospital | Central area | 2015 | 2011-2014 | 2011-2017 |
| Wuchang Hospital of Wuhan | Central area | 2015 | 2011-2014 | 2011-2017 |
| Huarun WISCO General Hospital Affiliated to Wuhan University of Science and Technology | Central area | 2015 | 2011-2014 | 2011-2017 |
| Wuhan Puren Hospital | Central area | 2015 | 2011-2014 | 2011-2017 |
| The First People's Hospital of Jiangxia District | Outlying area | 2015 | 2011-2014 | 2011-2017 |
| Maternal and Child Health Care Hospital of Jiangxia District of Wuhan | Outlying area | 2015 | 2011-2014 | 2011-2017 |
| Huangpi District People's Hospital | Outlying area | 2016 | - | 2015-2017 |

Table S2. Parental education level and occupation prestige rating scale

| Variable | Classification | SES Score | |
| --- | --- | --- | --- |
|  |  | Male | Female |
| Education | Doctor (22 years of education) | 69 | 73 |
|  | Master (19 years of education) | 69 | 73 |
|  | College (16 years of education) | 63 | 66 |
|  | Junior school (15 years of education) | 61 | 63 |
|  | High school (12 years of education) | 52 | 53 |
|  | Middle school (9 years of education) | 45 | 44 |
|  | Elementary school (6 years of education) | 36 | 34 |
|  | None | 29 | 28 |
| Occupation | Managerial workers | 59 | |
|  | Professionals & technical | 63 | |
|  | Office clerks | 56 | |
|  | Service workers | 46 | |
|  | Agriculture and forestry | 34 | |
|  | Laborer | 42 | |
|  | Soldier | 53 | |
|  | Others | 49 | |

SES: socioeconomic status.

Table S3. Living area specified parental SES and accessary to BFH

| Variables | Class | Urban area  [n (%), N =60,844] | Rural area  [n (%), N =37,625) | Total  (n, N=98,469) |
| --- | --- | --- | --- | --- |
| Maternal education^*^ | ≤9 y | 9,389 (25.3) | 27,795 (74.7) | 37,184 |
|  | 10-12 y | 7,342 (46.5) | 8,437 (53.5) | 15,779 |
|  | 13-15 y | 11,012 (67.9) | 5,216 (32.1) | 16,228 |
|  | ≥16 y | 18,503 (77.1) | 5,489 (22.9) | 23,992 |
| Paternal education^*^ | ≤9 y | 2,666 (13.3) | 17,402 (86.7) | 20,068 |
|  | 10-12 y | 9,221 (37.7) | 15,206 (62.3) | 24,427 |
|  | 13-15 y | 12,636 (66.3) | 6,409 (33.7) | 19,045 |
|  | ≥16 y | 21,723 (73.3) | 7,920 (26.7) | 29,643 |
| Maternal occupation^*^ | Low prestige | 19,813 (33.1) | 40,033 (66.9) | 59,846 |
|  | High prestige | 26,433 (79.3) | 6,904 (20.7) | 33,337 |
| Paternal occupation^*^ | Low prestige | 17,941 (31.8) | 38,551 (68.2) | 56,492 |
|  | High prestige | 28,305 (77.1) | 8,386 (22.9) | 36,691 |
| Parental occupation^*^ | Parental low prestige | 13,603 (27) | 36,804 (73) | 50,407 |
|  | Maternal low and paternal high prestige | 6,210 (65.8) | 3,229 (34.2) | 9,439 |
|  | Maternal high and paternal low prestige | 4,338 (71.3) | 1,747 (28.7) | 6,085 |
|  | Parental high prestige | 22,095 (81.1) | 5,157 (18.9) | 27,252 |
| Overall SES^*^ | Low | 5,187 (16.8) | 25,771 (83.2) | 30,958 |
|  | Medium | 17,111 (53.5) | 14,856 (46.5) | 31,967 |
|  | High | 23,948 (79.1) | 6,310 (20.9) | 30,258 |
| Birth at a baby-friendly hospital^*^ | No | 6,558 (17.3) | 31,357 (82.7) | 37,915 |
|  | Yes | 39,688 (71.8) | 15,580 (28.2) | 55,268 |

SES: socioeconomic status; BFH: baby friendly hospital.

^*^: *p* < 0.05.

Table S4. Living area specified association between SES and EBF at six months old

| Variable | Class | Urban area | | |  | Rural area | | |
| --- | --- | --- | --- | --- | --- | --- | --- | --- |
|  |  | Model 1^a^  OR (95% CI) | Model 2^b^  OR (95% CI) | Model 3^c^  OR (95% CI) |  | Model 1^a^  OR (95% CI) | Model 2^b^  OR (95% CI) | Model 3^c^  OR (95% CI) |
| Maternal education | ≤9 y | REF | REF | REF |  | REF | REF | REF |
|  | 10-12 y | 0.72 (0.68, 0.77) | 0.74 (0.69, 0.79) | 0.74 (0.69, 0.79) |  | 1.34 (1.23, 1.47) | 1.34 (1.22, 1.46) | 1.21 (1.11, 1.33) |
|  | 13-15 y | 0.72 (0.68, 0.76) | 0.73 (0.68, 0.77) | 0.73 (0.69, 0.77) |  | 1.43 (1.28, 1.59) | 1.39 (1.25, 1.55) | 1.13 (1.01, 1.26) |
|  | ≥16 y | 0.76 (0.72, 0.80) | 0.75 (0.71, 0.79) | 0.76 (0.72, 0.80) |  | 1.26 (1.13, 1.41) | 1.21 (1.08, 1.36) | 0.95 (0.85, 1.07) |
| Paternal education | ≤9 y | REF | REF | REF |  | REF | REF | REF |
|  | 10-12 y | 1.09 (0.99, 1.20) | 1.09 (0.99, 1.20) | 1.10 (1.00, 1.21) |  | 1.43 (1.30, 1.56) | 1.41 (1.29, 1.54) | 1.26 (1.15, 1.38) |
|  | 13-15 y | 1.07 (0.98, 1.18) | 1.07 (0.97, 1.17) | 1.09 (0.99, 1.19) |  | 1.59 (1.43, 1.77) | 1.56 (1.40, 1.74) | 1.25 (1.12, 1.40) |
|  | ≥16 y | 0.99 (0.90, 1.08) | 0.97 (0.89, 1.07) | 0.99 (0.91, 1.09) |  | 1.43 (1.29, 1.59) | 1.40 (1.25, 1.56) | 1.09 (0.97, 1.22) |
| Maternal occupation | Low prestige | REF | REF | REF |  | REF | REF | REF |
|  | High prestige | 1.13 (1.08, 1.17) | 1.12 (1.07, 1.16) | 1.13 (1.08, 1.18) |  | 1.41 (1.29, 1.54) | 1.36 (1.24, 1.49) | 1.03 (0.94, 1.13) |
| Paternal occupation | Low prestige | REF | REF | REF |  | REF | REF | REF |
|  | High prestige | 1.16 (1.12, 1.21) | 1.15 (1.10, 1.20) | 1.16 (1.11, 1.21) |  | 1.52 (1.40, 1.65) | 1.49 (1.37, 1.62) | 1.17 (1.07, 1.27) |
| Parental occupation | Parental low prestige | REF | REF | REF |  | REF | REF | REF |
|  | Maternal low and paternal high prestige | 1.02 (0.95, 1.09) | 1.02 (0.95, 1.09) | 1.03 (0.95, 1.12) |  | 1.56 (1.38, 1.76) | 1.54 (1.37, 1.74) | 1.38 (1.21, 1.58) |
|  | Maternal high and paternal low prestige | 0.92 (0.85, 1.00) | 0.93 (0.85, 1.01) | 0.93 (0.84, 1.02) |  | 1.27 (1.06, 1.52) | 1.26 (1.05, 1.50) | 1.05 (0.87, 1.28) |
|  | Parental high prestige | 1.18 (1.12, 1.23) | 1.16 (1.11, 1.22) | 1.22 (1.15, 1.30) |  | 1.54 (1.39, 1.70) | 1.49 (1.35, 1.65) | 1.09 (0.98, 1.23) |
| Overall SES | Low | REF | REF | REF |  | REF | REF | REF |
|  | Medium | 1.00 (0.94, 1.07) | 1.01 (0.94, 1.07) | 1.01 (0.94, 1.07) |  | 1.42 (1.31, 1.53) | 1.40 (1.30, 1.52) | 1.23 (1.13, 1.34) |
|  | High | 0.91 (0.86, 0.97) | 0.90 (0.84, 0.96) | 0.90 (0.84, 0.96) |  | 1.47 (1.32, 1.62) | 1.43 (1.28, 1.59) | 1.06 (0.94, 1.19) |

CI: confidence interval; OR: odds ratio; SES: socioeconomic status.

^a^Model 1 used a logistic model without covariates.

^b^Model 2 used a logistic model with covariates including maternal childbearing age, maternal pre-pregnancy BMI, gestational weight gain, infant birth weight, birth mode, and sex.

^c^Model 3 used a logistic model with covariates including variables adjusted in model 2 and birth at a baby-friendly hospital.

Table S5. Living area specified association between SES and birth at a baby-friendly hospital

| Variable | Class | Urban area | |  | Rural area | |
| --- | --- | --- | --- | --- | --- | --- |
|  |  | Model 1^a^  OR (95% CI) | Model 2^b^  OR (95% CI) |  | Model 1^a^  OR (95% CI) | Model 2^b^  OR (95% CI) |
| Maternal education^#^ | ≤9 y | REF | REF |  | REF | REF |
|  | 10-12 y | 0.99 (0.98, 1.01) | 0.99 (0.98, 1.01) |  | 1.38 (1.34, 1.44) | 1.35 (1.31, 1.40) |
|  | 13-15 y | 1.08 (1.07, 1.1) | 1.06 (1.05, 1.08) |  | 1.98 (1.91, 2.05) | 1.76 (1.71, 1.82) |
|  | ≥16 y | 1.15 (1.14, 1.17) | 1.11 (1.1, 1.13) |  | 2.26 (2.19, 2.33) | 1.88 (1.82, 1.94) |
| Paternal education^*^ | ≤9 y | REF | REF |  | REF | REF |
|  | 10-12 y | 1.15 (1.12, 1.19) | 1.14 (1.11, 1.17) |  | 1.56 (1.51, 1.62) | 1.50 (1.45, 1.55) |
|  | 13-15 y | 1.23 (1.2, 1.27) | 1.19 (1.16, 1.23) |  | 2.26 (2.17, 2.35) | 2.01 (1.93, 2.08) |
|  | ≥16 y | 1.31 (1.28, 1.34) | 1.25 (1.22, 1.28) |  | 2.49 (2.4, 2.58) | 2.14 (2.06, 2.21) |
| Maternal occupation^*^ | Low prestige | REF | REF |  | REF | REF |
|  | High prestige | 1.12 (1.11, 1.13) | 1.09 (1.08, 1.10) |  | 2.32 (2.26, 2.37) | 1.94 (1.89, 1.99) |
| Paternal occupation^*^ | Low prestige | REF | REF |  | REF | REF |
|  | High prestige | 1.11 (1.10, 1.12) | 1.08 (1.07, 1.09) |  | 2.16 (2.11, 2.22) | 1.86 (1.81, 1.9) |
| Parental occupation^*^ | Parental low prestige | REF | REF |  | REF | REF |
|  | Maternal low and paternal high prestige | 1.08 (1.07, 1.1) | 1.07 (1.05, 1.08) |  | 1.65 (1.58, 1.72) | 1.55 (1.48, 1.61) |
|  | Maternal high and paternal low prestige | 1.12 (1.11, 1.14) | 1.10 (1.08, 1.11) |  | 1.87 (1.78, 1.97) | 1.7 (1.62, 1.78) |
|  | Parental high prestige | 1.16 (1.15, 1.17) | 1.12 (1.11, 1.13) |  | 2.63 (2.57, 2.7) | 2.15 (2.1, 2.21) |
| Overall SES^*^ | Low | REF | REF |  | REF | REF |
|  | Medium | 1.13 (1.11, 1.15) | 1.11 (1.09, 1.13) |  | 1.74 (1.69, 1.79) | 1.64 (1.59, 1.69) |
|  | High | 1.25 (1.23, 1.27) | 1.20 (1.18, 1.22) |  | 2.73 (2.65, 2.82) | 2.25 (2.18, 2.32) |

BMI: body mass index; CI: confidence interval; BFH: baby-friendly hospital; NBFH: not baby-friendly hospital; OR: odds ratio; SES: socioeconomic status.

^#^: p values for partly models significant.

^*^: *p* values for all levels significant.

^a^Model 1 used a log-binomial model without covariates.

^b^Model 2 used a log-binomial model with covariates including maternal childbearing age, pre-pregnancy BMI, gestational weight gain and birth mode.

Table S6. Living area specified association between BFH and EBF

|  | Urban area | |  | Rural area | |
| --- | --- | --- | --- | --- | --- |
|  | Model 1^a^  OR (95% CI) | Model 2^b^  OR (95% CI) |  | Model 1^a^  OR (95% CI) | Model 2^b^  OR (95% CI) |
| Overall population^*^ | | | | | |
| Non-BFH | REF | REF |  | REF | REF |
| BFH | 0.89 (0.84, 0.94) | 0.91 (0.86, 0.96) |  | 2.51 (2.33, 2.7) | 2.45 (2.27, 2.64) |
| Low SES group^*^ | | | | | |
| Non-BFH | REF |  |  | REF |  |
| BFH | 1.04 (0.91, 1.19) |  |  | 3.16 (2.84, 3.52) |  |
| Medium SES group^*^ | | | | | |
| Non-BFH | REF |  |  | REF |  |
| BFH | 0.86 (0.79, 0.93) |  |  | 1.88 (1.67, 2.12) |  |
| High SES group^*^ | | | | | |
| Non-BFH | REF |  |  | REF |  |
| BFH | 0.9 (0.82, 0.99) |  |  | 2 (1.62, 2.48) |  |

^*^: *p* values for all levels significant.

^a^ Model 1 used a log-binomial model with covariates including maternal childbearing age, pre-pregnancy BMI, gestational weight gain, birth mode, infants’ gender and birth weight.

^b^ Model 2 used a log-binomial model with covariates including maternal childbearing age, pre-pregnancy BMI, gestational weight gain, birth mode, infants’ sex, birth weight and overall socioeconomic status.

Table S7. Living area specified mediation effect of birth at a baby-friendly hospital in the association between familial socioeconomic status and infant exclusive breastfeeding at six months old.

| Living area | Groups^a^ | Direct effect [log (RR)] | |  | Indirect effect[log (RR)] | | Total effect[log (RR)] |
| --- | --- | --- | --- | --- | --- | --- | --- |
|  |  | Pure | Total |  | Pure | Total |  |
| Urban area | Medium vs Low | 0.02 (0.03) | -0.01 (0.03) |  | 0.01 (0.01) | -0.01 (0.01) ^*^ | 0.01 (0.03) |
|  | High vs Low | -0.09 (0.04) ^*^ | -0.12 (0.04) ^*^ |  | 0.01 (0.01) | -0.02 (0.01) ^*^ | -0.11 (0.04) ^*^ |
| Rural area | Medium vs Low | 0.24 (0.04) ^*^ | 0.14 (0.04) ^*^ |  | 0.20 (0.01) ^*^ | 0.10 (0.01) ^*^ | 0.43 (0.04) ^*^ |
|  | High vs Low | 0.14 (0.07) ^*^ | -0.04 (0.06) |  | 0.40 (0.02) ^*^ | 0.22 (0.04) ^*^ | 0.36 (0.05) ^*^ |

^a^Covariates in the mediation model included maternal childbearing age, pre-pregnancy BMI, gestational weight gain, infant birth weight, birth mode, and sex.

^*^refers to *p* <0.005 for all levels.

The direct and indirect effects for all the comparing groups were significant among the direct (both pure and total effect), indirect (both pure and total effect) and total effects.

Table S8. Effect s of co-variables in the association analyses of SES-EBF, BFH-EBF, and SES-BFH.

|  | SES-EBF model | | BFH-EBF model | | SES-BFH model | |
| --- | --- | --- | --- | --- | --- | --- |
|  | OR (95% CI) | *P* value | OR (95% CI) | *P* value | OR (95% CI) | *P* value |
| Maternal pre-pregnancy BMI | | | | | | |
| Underweight | REF |  | REF |  | REF |  |
| Normal | 1.16 (1.11, 1.21) | < 0.001 | 1.15 (1.1, 1.2) | < 0.001 | 1.01 (1, 1.02) | < 0.01 |
| Overweight/  Obesity | 1.22 (1.13, 1.31) | < 0.001 | 1.19 (1.11, 1.28) | < 0.001 | 1.02 (1.01, 1.04) | < 0.01 |
| Gestational weight gain | | | | | | |
| Excessive | REF |  | REF |  | REF |  |
| Inadequate | 1.1 (1.02, 1.19) | 0.01 | 1.09 (1.02, 1.18) | 0.02 | 1 (0.99, 1.02) | 0.62 |
| Appropriate | 1.07 (1.03, 1.11) | < 0.001 | 1.07 (1.03, 1.11) | < 0.001 | 1.01 (1, 1.01) | 0.11 |
| Maternal childbearing age | | | | | | |
| <25 y | REF |  | REF |  | REF |  |
| 25-<30 y | 1.11 (1.05, 1.16) | < 0.001 | 1.02 (0.97, 1.08) | 0.35 | 1.3 (1.28, 1.33) | < 0.001 |
| 30-<35 y | 1.2 (1.14, 1.28) | < 0.001 | 1.06 (1, 1.13) | 0.04 | 1.41 (1.38, 1.44) | < 0.001 |
| ≥35 y | 1.41 (1.29, 1.55) | < 0.001 | 1.21 (1.11, 1.32) | < 0.001 | 1.3 (1.28, 1.33) | < 0.001 |
| Birth mode | | | | | | |
| Transvaginal | REF |  | REF |  | REF |  |
| Cesarean | 0.78 (0.75, 0.81) | < 0.001 | 0.84 (0.81, 0.87) | < 0.001 | 0.89 (0.88, 0.9) | < 0.001 |
| Infant birth weight | | | | | | |
| <2500 g | REF |  | REF |  |  |  |
| 2500-4000 g | 1.01 (0.85,1.21) | 0.90 | 1.01 (0.84, 1.21) | 0.91 |  |  |
| ≥4000 g | - 1. (1,1.17) | 0.04 | 1.06 (0.98, 1.14) | 0.13 |  |  |
| Sex | | | | | | |
| Girl | REF |  | REF |  |  |  |
| Boy | 0.96 (0.92, 0.99) | 0.01 | 0.96 (0.93, 0.99) | 0.02 |  |  |

In the SES-EBF model, the outcome variable was EBF and the independent variable was SES.

In the BFH-EBF model, the outcome variable was EBF and the independent variable was BFH with SES also adjusted for.

In the SES-BFH model, the outcome variable was BFH and the independent variable was SES with infant birth weight and sex not adjusted for


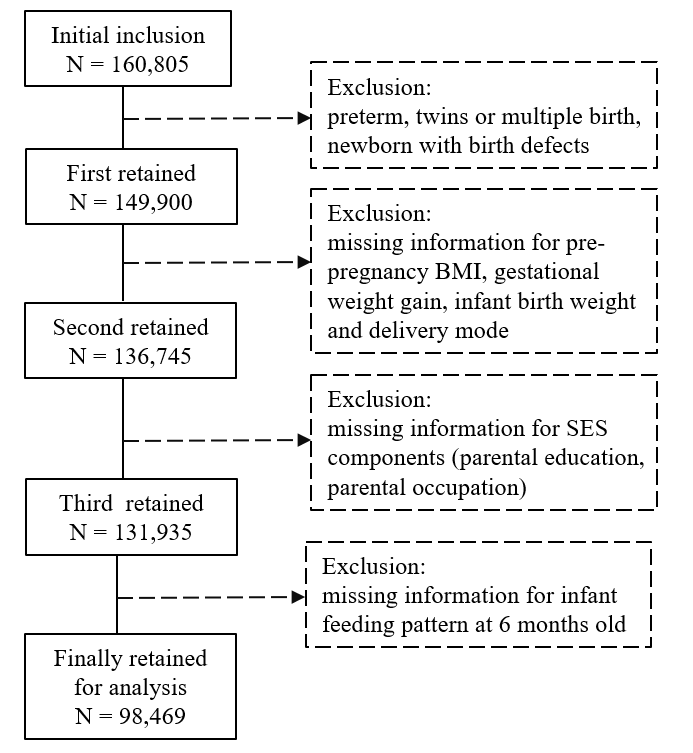


Figure S1. Flowchart for participants’ inclusion
